# Unraveling novel insights into dual-species cariogenic biofilm formation on aged teeth: a comparative analysis on natural vs artificial bioengineered dentin models

**DOI:** 10.1128/aem.01721-25

**Published:** 2025-10-30

**Authors:** Javiera Ortiz, Simón Álvarez, Sebastian Aguayo

**Affiliations:** 1 Institute for Biological and Medical Engineering, Schools of Engineering, Medicine and Biological Sciences, Pontificia Universidad Católica de Chile 28033, Santiago, Chile; 2 School of Medicine, Faculty of Medicine, Pontificia Universidad Católica de Chile 28033, Santiago, Chile; 3 Dentistry School, Faculty of Medicine, Pontificia Universidad Católica de Chile 28033, Santiago, Chile; Indiana University Bloomington, Bloomington, Indiana, USA

**Keywords:** *Streptococcus mutans*, *Candida albicans*, microfabrication, collagen, bacterial adhesion, organ on a chip

## Abstract

Dental caries is the most prevalent biofilm-associated disease affecting billions of people worldwide, including elderly individuals. Conventional biofilm study methods rely on human or animal-derived samples, posing challenges regarding accessibility, cost, and ethical considerations. While *in vitro* systems offer a promising alternative, they often fail to replicate the structural characteristics of dentin, which play a crucial role in bacterial adhesion. To bridge this gap, a bioengineered dentin construct has recently been developed as a reproducible and accessible model for studying biofilm formation specifically associated with dental aging. Therefore, this study aimed to assess dual-species *Streptococcus mutans* and *Candida albicans* biofilm formation on bioengineered dentin substrates and compare it to biofilm formation on natural human aged dentin. For this, *S. mutans* UA159 and *C. albicans* (ATCC 90028) were co-cultured on bioengineered and natural dentin slabs, and polymicrobial biofilm formation and extracellular polysaccharide matrix production were characterized via high-resolution confocal laser scanning microscopy. Following biofilm formation, image processing was conducted using COMSTAT software to determine biofilm growth parameters. Additionally, fluorescence intensity was quantified via microplate readings, and cell viability was assessed using a Live/Dead viability kit. Overall, results showed comparable biofilm formation patterns between the bioengineered and aged dentin, with no significant differences found in biofilm physical properties or viability. These findings suggest that this bioengineered dentin construct provides a reliable platform for studying biofilm formation in the context of dental aging, making it a valuable tool for investigating microbial adhesion and cariogenic biofilm development under controlled conditions, potentially facilitating future research in biofilm-related oral diseases.

**IMPORTANCE:**

Dental caries is one of the most common chronic diseases worldwide and is driven by complex microbial biofilms formed on the tooth’s surface. However, existing models for studying these biofilms in the laboratory often rely on human or animal tissues, which are difficult to standardize and present ethical challenges. In this study, we validate a bioengineered dentin-like model that accurately mimics the microarchitecture of aged human dentin, a key site for root caries in the elderly. By comparing biofilms formed by the clinically significant *Streptococcus mutans* and *Candida albicans* on both artificial and natural substrates, we show that the engineered model supports biofilm development under comparable parameters and enables detection of changes in microbial virulence. Overall, this platform provides a reproducible and scalable alternative for studying oral biofilms with potential applications in understanding disease pathogenesis, novel treatment testing, and integration into next-generation organ-on-a-chip systems.

## INTRODUCTION

Caries is the most prevalent disease worldwide, affecting over 2 billion people (1). With a strong tendency toward aging populations (2, 3), it remains necessary to continue promoting long-lasting oral health and developing specifically tailored preventive and therapeutic measures (2–4). In this context, understanding how host-pathogen interactions at the dental interface can lead to the development of caries is crucial to unveil new strategies. To date, the most employed experimental methods for studying oral biofilm formation under controlled laboratory conditions are *in vitro* studies, animal models, and the use of *ex vivo* human dentin samples (5–7); however, these approaches can be costly, and the acquisition of animal or human samples may present significant bioethical and experimental challenges.

Currently, microfabricated and microfluidic devices enable dynamic *in vitro* experiments with reduced costs and have been successfully applied to study the pathophysiology of various organs such as the heart, liver, and others (8–10). Building on this technology, “*tooth-on-a-chip*” models have been developed to investigate microbial, cellular, and biomaterial interactions by simulating different oral environments (11). One key aspect that can be studied using these models is biofilm adhesion (12); however, existing tooth-on-a-chip models still lack the incorporation of a critical factor for biofilm formation on dentin: substrate microtopography. Furthermore, while many static *in vitro* models examine biofilm adhesion on hydroxyapatite disks that mimic the smooth enamel tissue surface, dentin tissue remains largely unexplored. This is particularly relevant considering that dentin is volumetrically more abundant than enamel in natural teeth (13). This imbalance becomes even more pronounced with aging, as enamel progressively wears down due to physiological processes such as attrition, abrasion, and erosion. It is estimated that enamel thickness in teeth of older individuals (aged over 65 years) is reduced by approximately one-third compared with newly erupted teeth (14), making dentin a relevant substrate for studying biofilm development, especially in older populations, in the context of coronal and root caries.

Dentin is a tissue that has a tubular microarchitecture and is composed mostly of inorganic hydroxyapatite (HAP) but also around 20% of a type-I collagen organic matrix (15). Dentin exposure occurs not only due to this age-related enamel loss but also due to pathological or mechanical factors such as caries, trauma, or gingival recession, which is estimated to affect two-thirds of the global population, with higher prevalence among older adults (16). Most importantly, dentin exposure allows colonization by resident oral biofilms and increases the risk of deep caries and root caries, which can lead to the loss of tooth vitality (17, 18). Although the composition of dentin and its interaction with adhesive, restorative, or regenerative biomaterials are widely studied topics in dentistry, the influence of dentin architecture on microbial adhesion and subsequent biofilm formation is rarely considered in existing models (19).

*Streptococcus mutans*, one of the most studied cariogenic species (20), initiates colonization by adhering to the acquired pellicle—a salivary protein layer composed of mucins, agglutinins, and proline-rich proteins (PRPs)—via the interaction of specific adhesins (21). However, *S. mutans* can also bind directly to tissue collagen through adhesins like Cnm, Cbm, SpaP, and WapA (22, 23). In the presence of sucrose, it produces extracellular glucans via glucosyltransferases (e.g., GtfB), contributing to exopolysaccharide (EPS) matrix formation, which enhances biofilm development and stability (24). Additionally, *S. mutans* also exhibits a synergistic relationship with *Candida albicans* (25, 26), and together, they are linked to rapidly progressing caries in both infants and elderly root caries (27). *C. albicans* is an opportunistic fungus and common colonizer of the oral cavity present in 30%–35% of healthy adults and up to ~75% in elderly populations (28, 29). Its prevalence further increases among prosthodontic wearers 60%–75%, which is frequently associated with denture stomatitis (30). Although *C. albicans* is not considered strongly cariogenic on its own, it plays a critical role in mixed-species biofilms. Its ability to enhance biofilm biomass, increase acid production, and promote tissue invasion—especially in synergy with *S. mutans*—supports its relevance as a key microbial contributor to the cariogenic process (31). With advancing age, the risk of developing root caries increases (32), and so does the prevalence of *C. albicans* (33). Notably, it has been isolated from dental plaque in root caries lesions in individuals over 55 years of age and positively correlated with the abundance of *S. mutans* (34). While no study has directly linked age, root caries, and *Candida* colonization in a unified model, the convergence of these factors suggests a plausible synergistic role of *C. albicans* in root caries pathogenesis within aging-related biofilm dysbiosis.

Furthermore, the accumulation of advanced glycation end products during aging has been associated with changes in collagen structure, as they act as cross-links between fibers and lead to alterations in the mechanical properties of collagen-rich tissues (35). In the specific context of dentin, this phenomenon results in increased stiffness of the collagen matrix (36). Moreover, recent studies on bacterial adhesion to glycated dentin have shown enhanced biofilm adhesion and growth, which could partially explain the differences in dysbiosis observed in elderly and individuals with diabetes (37–39).

To address the lack of studies on bacterial adhesion to dentin, particularly in aged teeth, and to integrate this research into microfluidic devices, a bioengineered dentin construct mimicking the tubular architecture of dentin was recently developed by our group (40). These constructs can be experimentally glycated to simulate tooth aging, providing consistent and predictable substrates that ensure reproducible experimental results. Furthermore, its scaled-down composition reduces costs associated with reagent use and addresses the potential ethical concerns related to the acquisition and handling of human and animal materials.

Despite these experimental advances, the ability of these bioengineered constructs to allow the growth and development of oral biofilms, with a composition and structure comparable to those grown on natural older teeth, remains unclear. Therefore, the aim of this study was to compare early cariogenic biofilm formation on aged artificial dentin and aged natural human dentin in a sucrose-enriched environment, by employing clinically relevant dual-species *S. mutans* and *C. albicans* biofilm.

## MATERIALS AND METHODS

### Sample collection and experimental design

An *in vitro* experimental study was conducted to evaluate biofilm formation on bioengineered and natural dentin substrates. Briefly, dual-species biofilms of *C. albicans* (ATCC 90028) and *S. mutans* UA 159 were cultured for 24 h in a 1:1 mixture of Brain Heart Infusion (BHI) and Tryptic Soy Broth (TSB) supplemented with 1% sucrose, at 37°C and 5% CO_₂_. Microbial concentrations were standardized using OD_630_ values calibrated against CFU/mL via reference curves. Equal volumes (50 µL each) of *S. mutans* (2.3 × 10^8^ CFU) and *C. albicans* (2.6 × 10^7^ CFU) suspensions were combined to optimize dual-species growth, and 100 µL of the mixture was inoculated per substrate.

The natural dentin slabs were collected from extracted caries-free teeth donated by adult patients (over 50 years old), from extractions performed for clinical reasons (e.g., poor prognosis or planned prosthetic treatment) under informed consent and approval by the Institutional Ethics Committee of the Pontificia Universidad Católica de Chile (approval ID: 210615001). Briefly, caries-free teeth were selected and cleaned with 70% ethanol. Then, 300 µm thick cross sections were obtained at the root level with a hard tissue microtome (Leica SP1600, USA). Approximately 20 slabs were collected per tooth and disinfected using 70% ethanol, sonication, and UV exposure for 15 min. The dentin slabs were manually prepared to approximately 5 mm in diameter, and their size was verified with a periodontal probe. Bioengineered dentin substrates were constructed and glycated with methylglyoxal (MGO) as previously described (40). They were standardized to 5- mm diameter using a biopsy punch and fabricated from the same mold to ensure uniform surface area and topography. These substrates were plasma-cleaned (Diener electronic, Zepto), and all subsequent functionalization steps were performed under aseptic conditions.

### Biofilm formation on tooth and PDMS substrates

**Fig 1:**
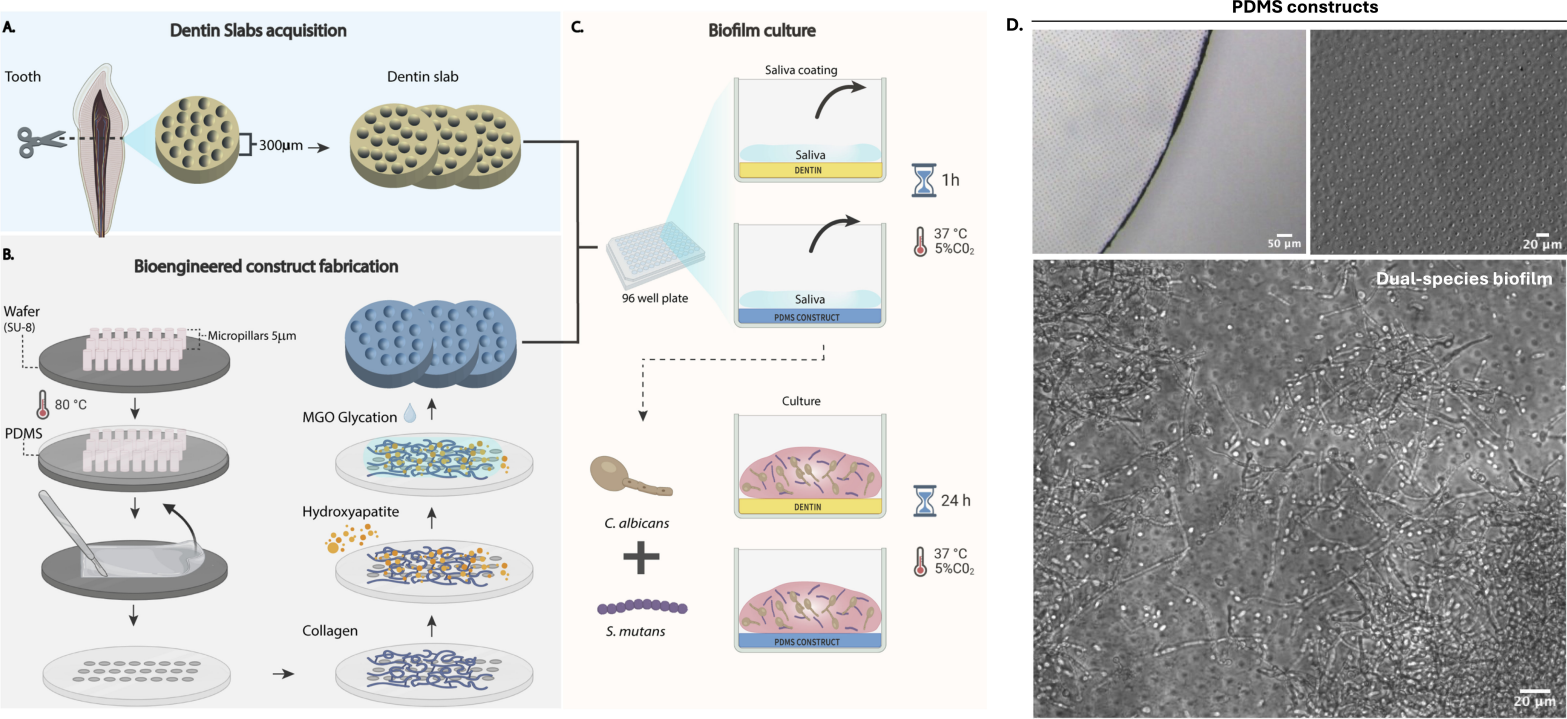
Diagrammatic representation of the experimental setup regarding construction of the bioengineered dentin substrates and preparation of natural dentin specimen. (**A**) Tooth acquisition with cross-sectional cut; (**B**) Bioengineered dentin construct fabrication with PDMS and functionalization; (**C**) Saliva coating and substrate culture with *S. mutans* and *C. albicans*. (**D**) Optical microscopy images of bioengineered dentin substrates (upper panels), and epifluorescence microscopy of a dual-species *C. albicans* and *S. mutans* biofilm grown on the biomaterial substrate (lower panel).

Substrates were placed individually in sterile 96-well plates. In all experiments, substrates were preconditioned with artificial saliva before inoculation, except one specific comparison evaluating its effects (with vs without treatment). For saliva preconditioning, 100  µL of artificial saliva (Sigma-Aldrich) was applied onto each substrate, incubated for 1 h at room temperature, and removed before seeding. Then, 100  µL of the dual-species inoculum (50 µL of each species) was applied directly onto the substrates. Plates were incubated under static conditions at 37°C and 5% CO_₂_ for 24 h. After incubation, non-adherent cells were gently removed by rinsing with sterile phosphate-buffered saline (PBS). Uninoculated substrates were included as negative controls to confirm sterility. The experimental setup is illustrated in Fig. 1.

### Biofilm viability assessment

To determine viability following surface growth, biofilms were stained using the LIVE/DEAD BacLight Bacterial Viability Kit (Molecular Probes, USA), which employs a combination of 1.67 µM Syto 9 and propidium iodide (PI) to differentiate live and dead/damaged bacteria, respectively. Stock solutions of PI (81845, Sigma; 20 mM) and SYTO 9 (S-34854, Invitrogen, Thermo Fisher Scientific; 3.34 mM) were prepared and used according to the BacLight kit protocol. A 1:1 mixture of stains in PBS was prepared to achieve final concentrations of 30 µM PI and 5 µM SYTO 9. The stain mixture was added to cells harvested from surfaces by sonication (*n* = 9). The samples were then incubated for 15 min in the dark at room temperature. As a control for dead staining, cells were treated with 70% ethanol for 1 hour prior to staining. After incubation, the supernatant was transferred to a 96-well microplate for spectrophotometric analysis (Synergy HT, Biotek).

### High-resolution confocal fluorescence microscopy imaging of biofilms

The organization and structural architecture of the biofilms were assessed using simultaneous *in situ* labeling of bacterial cells and EPS with confocal laser scanning microscopy (CLSM). In summary, 1 µM Alexa Fluor 647-labeled dextran conjugate (10,000 MW; Ex/Em: 647/668 nm; Molecular Probes, USA) was added to the culture medium during biofilm formation and development (1:1,000). After 24 h of incubation, excess stain was removed by washing once with PBS. The biofilms were then labeled with 2.5 µM SYTO 9 (Ex/Em: 488/516  nm; Molecular Probes, USA) in BHI (1:1,000) for 30 min, followed by Calcofluor White (Sigma M2R, 1  g/L + Evans blue 0.5  g/L; Ex/Em: 405/450  nm) in TSB (1:50) to stain fungal cells for 30 min. Careful PBS washes were performed after each staining step to remove excess reagent. CLSM imaging was conducted using a Zeiss LMS 880 AxioObserver with an Airyscan detector and 40×/1.2 NA water immersion objective. Pixel size was 0.0519 × 0.0519 µm, and optical section thickness was ~1  µm. Detection ranges were set to 450 nm (Calcofluor White), 516 nm (SYTO 9), and 654 nm (Alexa Fluor 647). Image acquisition, processing, and analysis were performed using ZEN Black 2.3 (ZEISS). Three samples were analyzed in triplicate; data were expressed as mean ± standard deviation. Statistical analyses were performed using GraphPad Prism Software 8.0 to compare differences between conditions.

### Quantification of biofilm parameters by COMSTAT

Following structural characterization, CLSM image stacks were analyzed using COMSTAT 2.1 (available at http://www.comstat.dk), a plugin for ImageJ designed to quantify morphometric parameters from confocal image stacks as previously described (41). Each Z-stack was separated by fluorescent channel, and each channel was analyzed individually. Key structural parameters, including total biomass, maximum thickness, roughness coefficient, and surface-to-biovolume ratio, were extracted for each channel. Before analysis, grayscale images were thresholded using automated thresholding methods (Otsu), selected per channel to best match the fluorescent signal distribution. The same thresholding approach was applied consistently across all samples for each channel to ensure comparability.

In addition to reporting individual values for each channel, mean values for total biomass, thickness, and roughness were calculated across all channels to provide a general overview of biofilm architecture. The surface-to-biovolume ratio was reported per channel due to its dependence on spatial distribution. Three-dimensional reconstructions were visualized using ZEN Blue Desk 2.3 (Zeiss) for qualitative assessment.

### Quantification of fluorophore proportions in dispersed biofilms using microplate fluorometry

To determine biofilm composition via fluorophore concentration after 24 h of culture, stained biofilms as previously described were harvested using an ISOLAB ultrasonic water cleaner (maximum power: 60 W). Briefly, only substrate samples with adherent biofilm were transferred to sterile Eppendorf tubes containing 300 µL of PBS, vortexed for 30 s, sonicated for 1 min, and vortexed again for 30 s (42). The resulting suspensions were then transferred in triplicate to a 96-well microplate for fluorophore concentration measurement using a microplate reader (Synergy HT, Biotek). A total of three independent experiments were analyzed, and the data were expressed as mean ± standard deviation. Statistical analyses were performed using GraphPad Prism 8.0 to compare differences between conditions.

### Quantitative RT-qPCR for virulence gene expression analysis

The expression of virulence-related genes in *S. mutans* and *C. albicans* was quantified using reverse transcription-quantitative polymerase chain reaction (RT-qPCR). Dual-species biofilms were cultured for 24 h, washed with PBS, and then detached by sonication as previously described. The detached biomass was used for subsequent RNA extraction, cDNA synthesis, and gene expression analysis. For *S. mutans*, we analyzed *spaP* (encoding adhesin P1, involved in collagen adhesion in the absence of sucrose), *gbpB* (encoding a glucan-binding protein involved in biofilm formation), and *gtfB* (encoding glucosyltransferase B, responsible for sucrose-dependent glucan synthesis and adherence). In *C. albicans*, we quantified the expression of ALS3 (encoding Als3, an adhesin involved in host cell interactions) and the expression of HWP1 (encoding Hwp1, a hyphal wall protein essential for hyphal formation and adhesion). The process involved RNA isolation, complementary DNA (cDNA) synthesis, and gene expression analysis via qPCR. Total RNA was extracted using the Monarch Total RNA Miniprep Kit (New England Biolabs), following the manufacturer’s instructions. Then, cDNA was synthesized from 10 µL of total RNA using the High-Capacity cDNA Reverse Transcription Kit (Applied Biosystem, US). The reaction mixture included 10 µL of master mix and was incubated in a thermocycler (QuantStudio 3, Thermo Fisher) under the following conditions: 25°C for 10 min, 37°C for 120 min, 85°C for 5 min, and a final hold at 4°C. Negative controls, consisting of RNA samples with all kit reagents except reverse transcriptase, were included to assess potential genomic DNA contamination. The resulting cDNA was stored at −20°C until further use.

Gene-specific primers for *S. mutans* and *C. albicans* were designed based on the *S. mutans* UA159 and *C. albicans* ATCC 90028 genome validated for specificity and efficiency (Table 1). Primer specificity was confirmed by melt curve analysis, and efficiency was determined using standard curves with target values of R² ≥ 0.99, efficiency between 90% and 110%, and a slope of approximately −3.3. Each 10 µL qPCR reaction contained 2 µL of cDNA, 8 µL of 2× SYBR Green Supermix (Thermo Fisher), with 1.4 µL of each primer, and molecular-grade water. Reactions were performed in triplicate using a QuantStudio three thermocycler with the following cycling conditions: initial denaturation at 95°C for 2 min, followed by 40 cycles of 95°C for 15 s, annealing at primer-specific temperatures (Table 1) for 20 s, and 72°C for 60 s (extension). A melt curve analysis was conducted at the end of the run to confirm the absence of primer-dimers and non-specific amplification. Amplification curves were analyzed using the QuantStudio Design and Analysis software. The threshold was set automatically by the software within the exponential phase of the amplification curves. The resulting Cq values were used for downstream analysis of gene expression levels.

**TABLE 1:** Primer design for *Streptococcus mutans* and *Candida albicans* strains^*a*^

Gene	Primer sequences (5′−3′)	Length, amplicon size (bp)	Annealing temp
16s	F: CCCGCATCAGATACTTGAGC R: ATACCCGCAACACAGGACTC	52	57°C
*spaP*	F: GAC TTT GGT AAT GGT TAT GCA TCA A R: TTT GTA TCA GCC GGA TCA AGT G	101	55°C
*gbpB*	F: ATG GCG GTT ATG GAC ACG TT R: TTT GGC CAC CTT GAA CAC CT	51	55°C
*gftB*	F: AGC AAT GCA GCC AAT CTA CAA AT R: ACG AAC TTT GCC GTT ATT GTC A	96	57°C
18s	F: GGATTTACTGAAGACTAACTACTG R: GAACAACAACCGATCCCTAGT	131	54°C
ALS3	F: CATTCGATCCTAACCGCG R: TGGGTTTGGCAGTGGAAC	108	56°C
HWP1	F: GCT CAA CTT ATT GCT ATC GCT TAT TAC A R: GACCGTCTACCTGTGGGACAGT	105	56°C

^*a*^ F, forward primer; R, reverse primer.

For *S. mutans* genes, relative expression was normalized to the 16s rRNA gene, and for *C. albicans* genes, 18s (a structural ribosome constituent) was selected as the normalizer, supported by their demonstrated stability under similar experimental conditions as reported in the literature (25, 43). Relative gene expression was calculated using the ΔΔCt method. Negative controls included no-template controls to rule out contamination.

## RESULTS AND DISCUSSION

### Bioengineered dentin substrates ensure biofilm viability during experimentation

**Fig 2:**
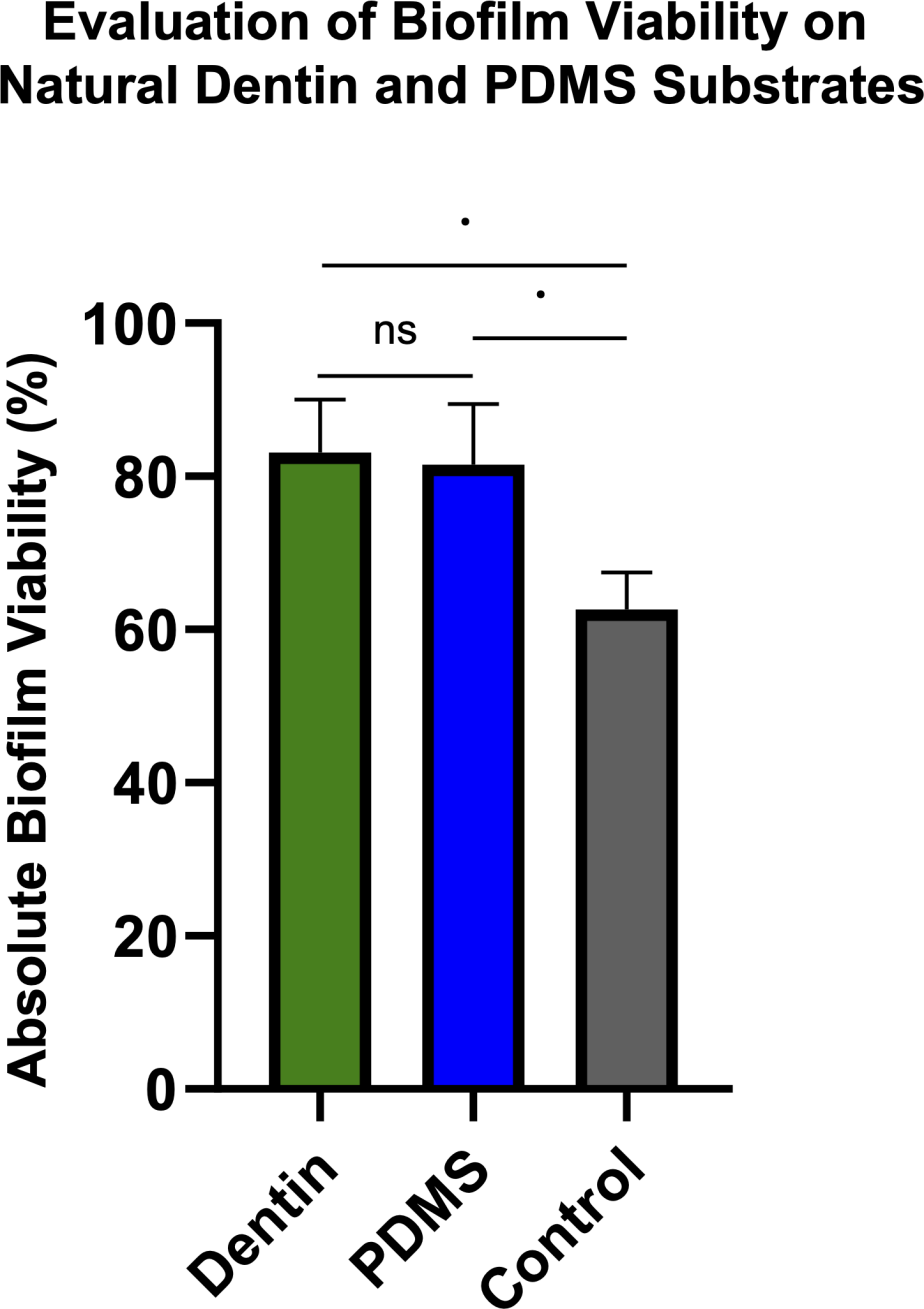
Cell viability (%) of dual-species *Streptococcus mutans* and *Candida albicans* biofilms on dentin and bioengineered dentin substrates, assessed using SYTO9 (live cells) and propidium iodide (dead cells) fluorescence after sonication-based detachment. Biofilms were grown for 24 h on a 96-well plate. Results are presented as mean values ± standard deviation (SD) from three independent experiments. Statistical analysis was performed using one-way ANOVA with Tukey’s post-hoc test. No statistically significant differences were found between substrates (*P* > 0.05). ns, non-significant.

First, to assess whether our bioengineered dentin construct supports bacterial viability comparably to natural dentin, we evaluated the proportion of live and dead cells after 24-h biofilm formation. No significant differences were observed between substrates, and both biofilms exhibited approximately 80% viability after 24-h growth (Fig. 2). The construct consisted of PDMS combined with HAP, collagen, and MGO, which was incorporated to simulate collagen aging through glycation. Although MGO is known to exert some cytotoxic effects on cells (44), its use for surface glycation previous to bacterial inoculation did not impair biofilm viability, probably due to the fact that all unreacted MGO is washed out of the system before biofilm experimentation. Thus, MGO-derived glycation of the construct can be performed without affecting subsequent biofilm viability and demonstrate the biocompatibility of the bioengineered construct to allow biofilm growth in a comparable manner to *ex vivo* dentin specimens.

### Biofilm formation on bioengineered constructs closely mimics growth on natural aged dentin

**Fig 3:**
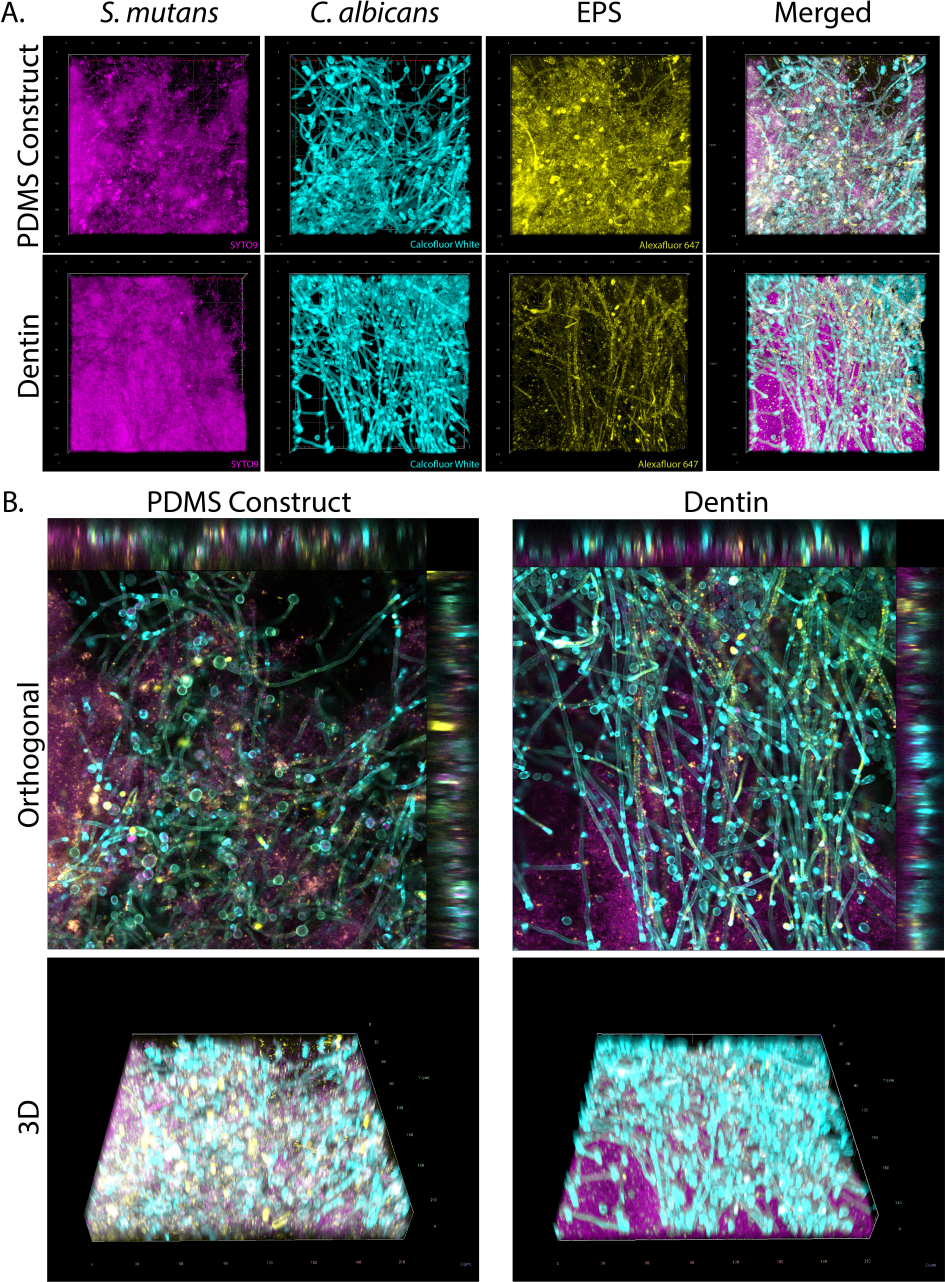
Confocal laser scanning microscopy (CLSM) images of dual-species biofilms cultured for 24 h on bioengineered dentin and natural dentin substrates. *S. mutans* bacterial microcolonies, stained with SYTO9, are shown in magenta, while fungal cells of *C. albicans*, stained with Calcofluor, are displayed in cyan. Extracellular polymeric substances (EPS), labeled with Alexa Fluor 647-dextran, are depicted in yellow. (**A**) 2D images of individual biofilm components, highlighting the spatial distribution of *S. mutans*, *C. albicans*, and EPS. (**B**) Orthogonal and three-dimensional reconstruction of the biofilm, demonstrating its structural complexity (40× magnification).

Following CLSM, images of the 24-h colonized substrates revealed a dense, multilayered, dual-species biofilm structure formed by *S. mutans* and *C. albicans* (Fig. 3). Adherence to both surfaces was characterized by a dense, compact biofilm, predominantly composed of tightly clustered *S. mutans* cells, primarily concentrated in the layer adjacent to the surface. Furthermore, *S. mutans* displayed the typical microcolony structure associated with local acidification of the biofilm, as expected, due to the increased sucrose supplementation in our system (45). *C. albicans* was predominantly observed in hyphal morphology across the z-axis, while EPS was dispersed in small clusters throughout the biofilm. Both substrates exhibited similar features (Fig. 3) and were comparable to biofilm structures observed on hydroxyapatite substrates, as reported in previous literature (46, 47).

**Fig 4:**
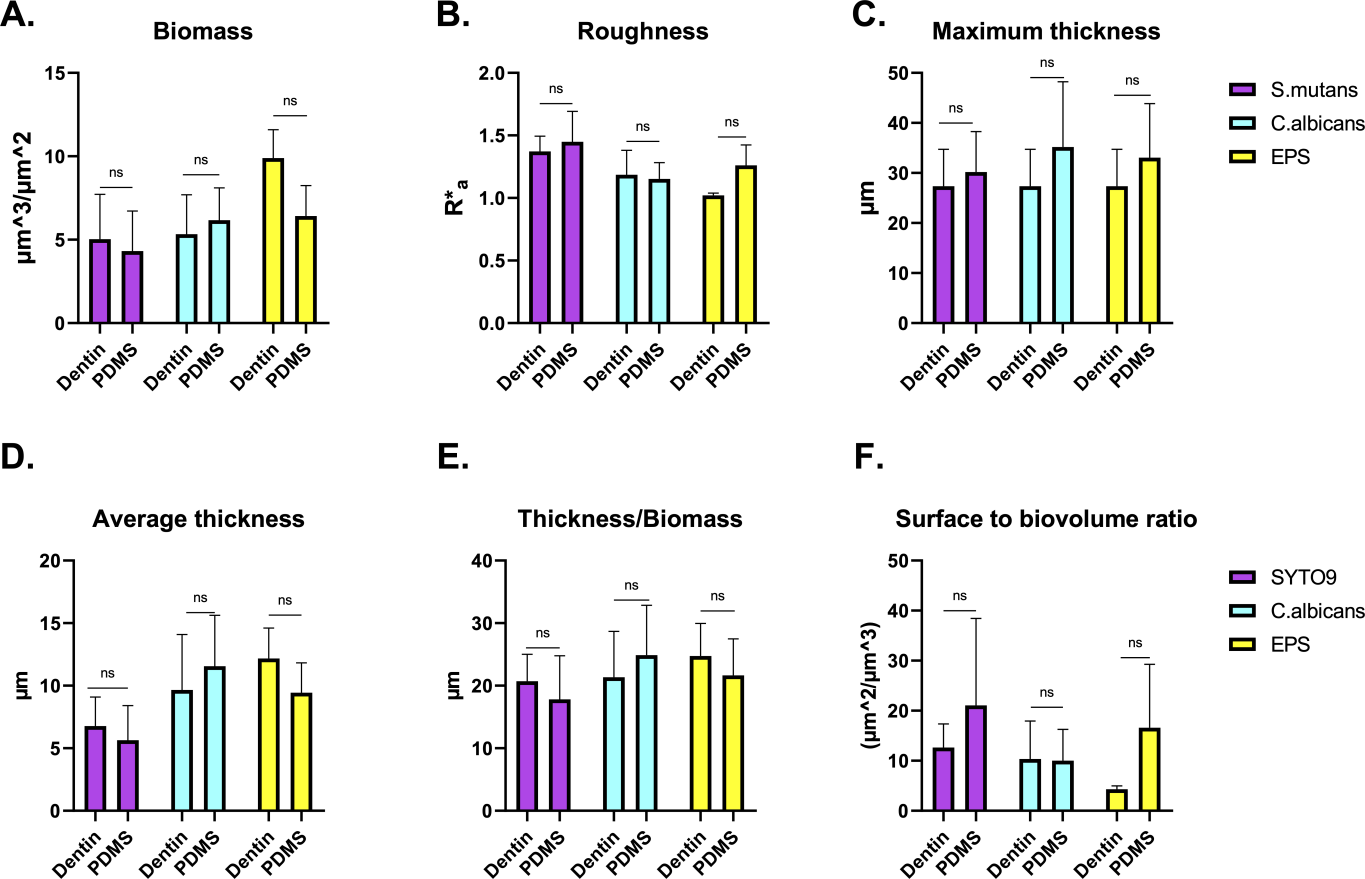
Quantitative evaluation of biofilm structural and roughness parameters. Bar graphs illustrate comparisons between ex-vivo dentin and bioengineered dentin substrates for total (**A**) biomass, (**B**) roughness, (**C**) maximum thickness, (**D**) average thickness, (**E**) thickness-to-biomass ratio, and (**F**) surface-to-biovolume ratio. Data are presented as mean values ± standard deviations, derived from a minimum of three independent experiments. Statistical analysis was conducted using *t*-tests and two-way ANOVA, followed by Tukey’s post hoc test. No statistically significant differences were found (*P*-value > 0.05). ns, non-significant.

Furthermore, quantitative analysis of the biofilm images was performed using COMSTAT to evaluate parameters, such as biomass, roughness, maximum thickness, average thickness, thickness-to-biomass ratio, and surface-to-biovolume ratio by channel. No significant differences were observed between groups in all the analyzed parameters, confirming the structural similarity of biofilms grown on natural and bioengineered dentin (Fig. 3 and 4). Maximum biofilm thickness averaged approximately 40 µm, which is expected given the short incubation times (24 h) that reflect early biofilm formation in our model (48). Regarding the analysis of individual channels, Calcofluor (*C. albicans*) was observed as the thickest component, whereas SYTO9 (*S. mutans*) represented the thinnest, with both substrates exhibiting similar patterns. This is expected as fungal cells are larger compared with streptococci, as well as having the ability to form hyphae that can extend well beyond 10 µm in length and adopt spatial configurations away from the surface (49, 50).

Although the quantitative parameters did not differ significantly between groups, a visual assessment of representative confocal images revealed apparent differences in the spatial distribution of species. In some samples, *S. mutans* tended to localize closer to the substrate in dentin discs, whereas in bioengineered dentin samples, it was more frequently observed toward the apical region of the biofilm. Conversely, *C. albicans* hyphae often extended throughout the vertical axis in both substrates, sometimes predominating in the upper biofilm layers, consistent with previous studies (31, 47, 51–53). These patterns, although not consistent across all replicates, suggest a degree of spatial reorganization influenced by substrate type. This may be partially attributed to subtle differences in surface properties between natural dentin and PDMS substrates. Although the engineered PDMS substrate was biofunctionalized with dentinal tubule-like features, collagen, and HAP to mimic natural dentin, it may still present a more homogeneous and controlled surface compared with the inherent heterogeneity of natural dentin. The presence of native dentinal tubules, mineral gradients, and residual organic matrix in human dentin could favor stronger basal adhesion of *S. mutans*, whereas the synthetic surface might allow greater vertical redistribution of microcolonies toward apical regions. This axial heterogeneity likely contributes to the high variability observed in surface-to-volume and thickness-related parameters (Fig. 4F) and may reflect physiological adaptation to the local microenvironment. Thus, understanding the influence of other physicochemical factors on biofilm microscale morphology and spatial organization should be the focus of future work.

On the other hand, a hallmark of oral biofilms is the presence of channels that allow the passage of water and nutrients while dispersing residual metabolites (54, 55). In this regard, all components showed a high thickness-to-biomass ratio, suggestive of a porous and dispersed biofilm architecture that is consistent with the presence of channels and areas of lower cellular and EPS density. Furthermore, the surface-to-biovolume analysis revealed greater variability in *S. mutans* and EPS, while *C. albicans* remained consistent across replicates. These findings suggest that *C. albicans* forms a structurally stable and robust framework within the biofilm, whereas *S. mutans* and EPS exhibit higher structural plasticity, likely reflecting adaptive responses to environmental or substrate-related conditions and associated with the formation of microcolony clusters (Fig. 4). This is supported by previous research that has found evidence that *C. albicans* can form a strong scaffold that allows the sustained growth and organization of *S. mutans* and other bacterial species within the oral biofilm (56–58). Overall, the present results confirm the structural complexity of dual *S. mutans-C. albicans* biofilms, which is acquired starting the initial stages of biofilm formation (≤ 24 h) with potential implications for late-stage biofilm characteristics (47).

### Combined CLSM and fluorometric analysis reveal stable biofilm composition across *ex vivo* and bioengineered dentin substrates

**Fig 5:**
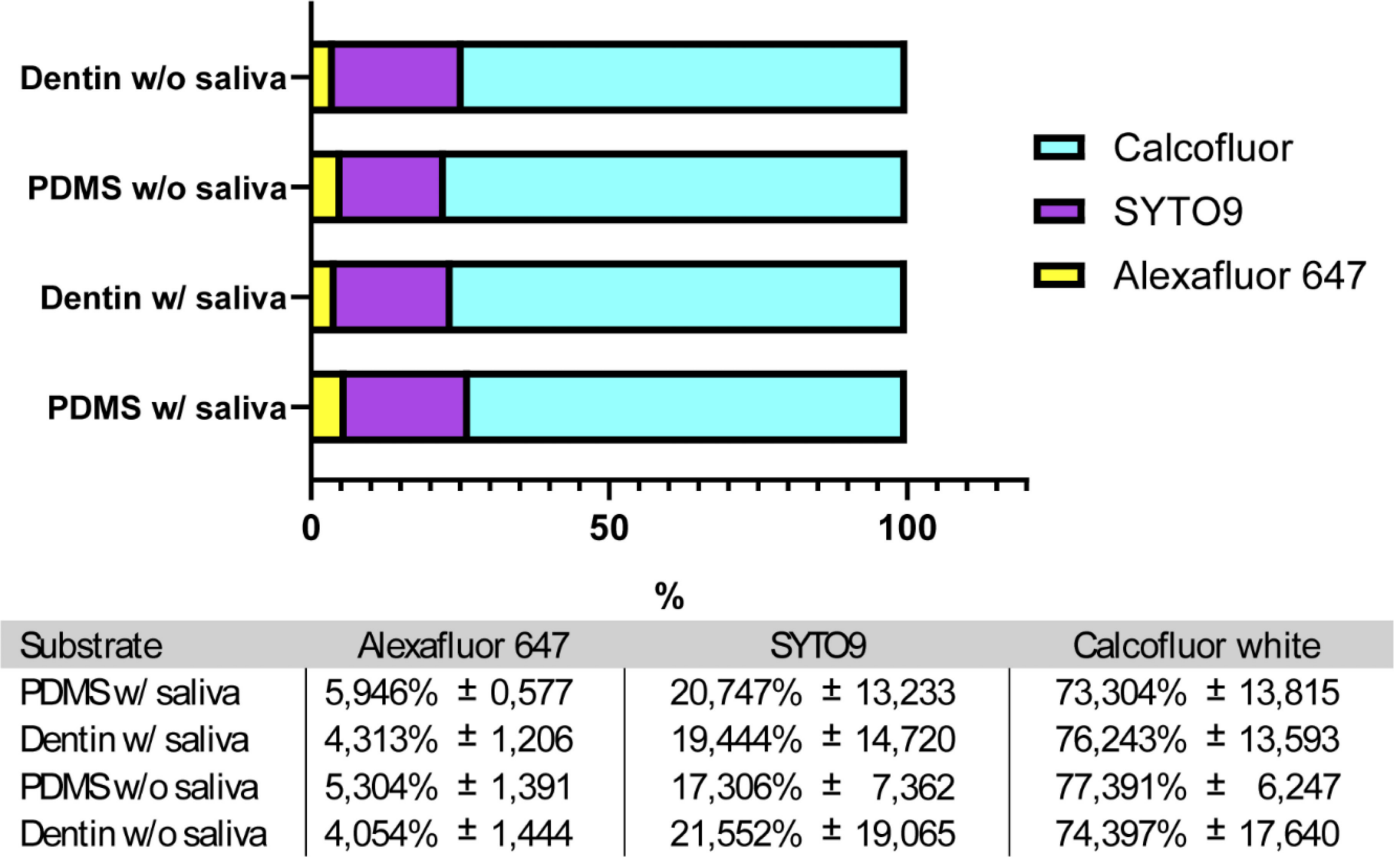
Proportions of relative fluorescence units (RFU) for SYTO9, Calcofluor, and Alexa Fluor 647, measured after sonication-based detachment of 24-h biofilms formed on dentin and bioengineered dentin substrate with or without saliva. Data are expressed as mean values ± standard deviation, calculated from three independent experiments. No statistically significant differences were found with (*P* > 0.05; two-way ANOVA followed by Tukey’s post-hoc test).

To further complement the CLSM observations, we quantified the relative proportion of fluorophores within the biofilm for each experimental condition using a microplate reader (Fig. 5). To do so, biofilms were detached, resuspended in PBS, and analyzed for fluorophore content. We also assessed whether the absence of salivary pre-coating would alter these proportions. Consistent with CLSM findings, Calcofluor was the most abundant fluorophore, followed by SYTO9 and, finally, Alexa Fluor. This suggests that the main biomass of the biofilm is dominated by *C. albicans*, followed by *S. mutans* and EPS. Furthermore, the EPS signal was found to be lower than the other biofilm components, closely matching the qualitative observations made from CLSM imaging. Most importantly, similar fluorophore proportions were observed across both substrates, with no significant differences between *ex vivo* and bioengineered dentin, confirming the relevance of the *in vitro* model to provide a relevant approximation of the *in vivo* setting. Additionally, the absence of saliva did not significantly impact the relative distribution of fluorophores within the biofilms (Fig. 5), suggesting that, despite being crucial during the initial bacteria-host interaction, the salivary pellicle only has a minor influence on polymicrobial biofilm composition and structure following 24 h of incubation (59).

### Environmental conditions impact cariogenic biofilm virulence on bioengineered dentin substrates: a proof of concept

**Fig 6:**
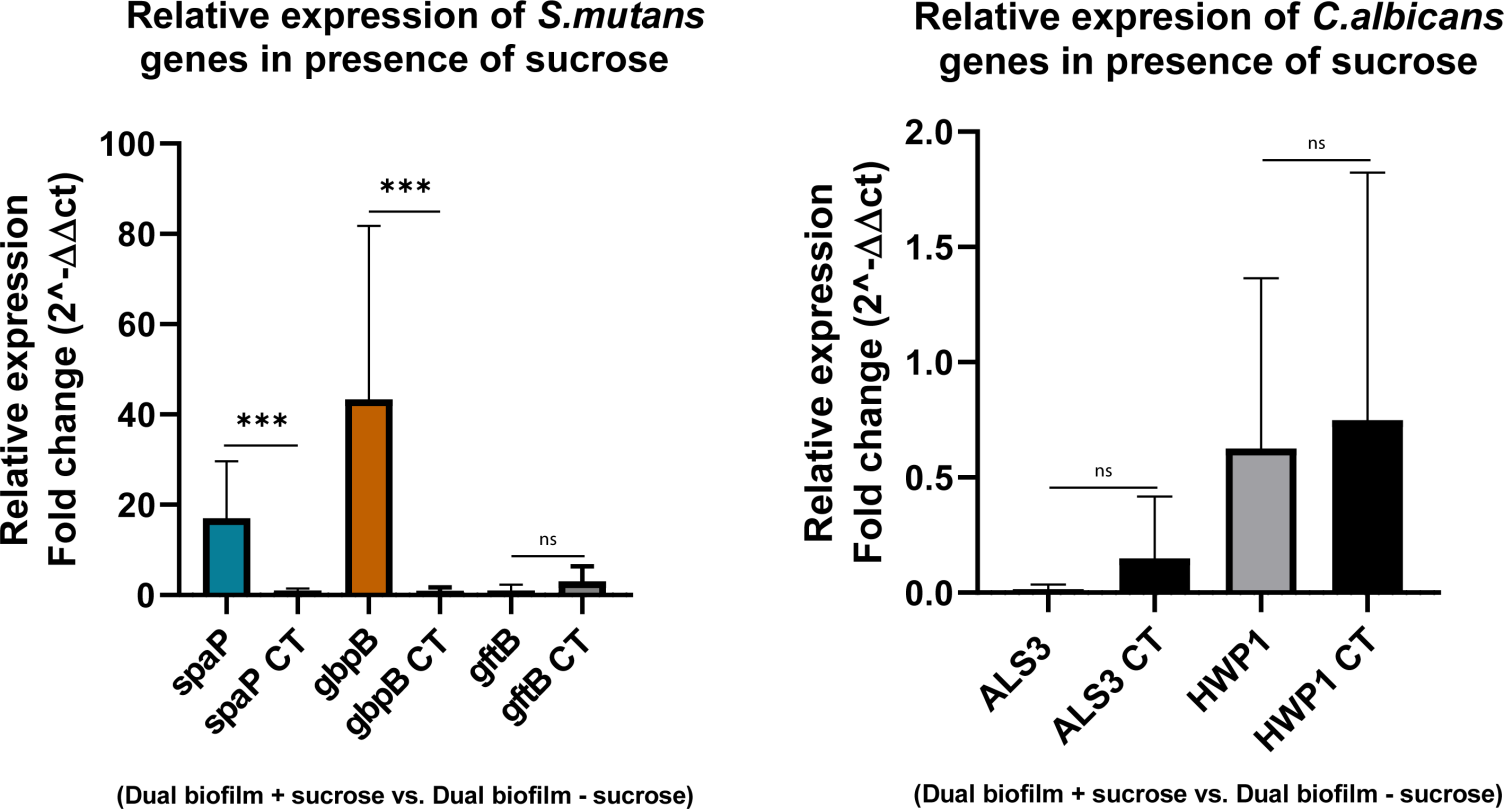
Expression of *S. mutans* UA159 genes (*spaP, gbpB, and gtfB*) and *Candida albicans* (*ALS3, HWP1*) analyzed by quantitative real-time PCR. (**A**) Fold change in *S. mutans* gene expression in a dual-species biofilm in the presence of sucrose. All data were normalized to the 16s housekeeping gene. (**B**) Fold change in *C. albicans* gene expression in the presence of sucrose. Bars labeled “CT” represent the control condition for each gene, used for ΔΔCt calculations. All data were normalized to the 18s housekeeping gene. Results represent at least three independent biological replicates, each performed in triplicate. Statistical analysis was performed using unpaired non-parametric Mann–Whitney U test. Statistically significant differences are indicated with asterisks (***); a *P*-value less than 0.05 was considered significant. ns, non-significant.

Once the functionality of the bioengineered dentin construct to harbor oral biofilms was established, we evaluated if the system allows for the assessment of biofilm virulence changes as a function of environmental conditions in a microscale assay (Fig. 6). For this, we compared the gene expression of key virulence genes associated with adhesion, biofilm formation, and EPS production in dual-species *S. mutans* and *C. albicans* biofilms grown in the presence and absence of supplemental sucrose.

Our results showed that the sucrose environment significantly upregulates *spaP*, a *S. mutans* gene associated with saliva and collagen binding (60), as well as *gbpB*, which is involved in glucan binding and has been linked to sucrose-enhanced metabolic activity (61). In contrast, *gtfB* was unexpectedly downregulated. We hypothesize that this may be due to the temporal dynamics of gene expression: *gtfB* may exhibit higher transcriptional activity during the exponential stages of biofilm development (4–6 h), while *gbpB* becomes more dominant at later stages (e.g., 24 h). This pattern aligns with previous *S. mutans* gene expression studies (47, 62). Additionally, reduced sucrose levels in the microenvironment could directly suppress *gtfB* expression, as this gene is highly responsive to sucrose concentration (63). In our experiment, the culture medium was not refreshed, which may have led to a rapid depletion of available sucrose and could partly explain the low EPS levels observed in the CLSM images after 24-h growth (Fig. 2).

Second, we evaluated *C. albicans* gene expression in the dual-species biofilm model and observed no significant differential expression in the presence versus absence of sucrose. This was expected for the target genes (HWP1, ALS3) as they are not directly sucrose responsive (64). However, we hypothesized that sucrose might indirectly modulate their expression through its known stimulatory effect on *S. mutans* gtfB-mediated glucan synthesis (46). The resulting extracellular glucan matrix was anticipated to serve as a surface signal for *C. albicans*, potentially enhancing hyphal development and upregulating hypha-associated adhesins through the MAPK/Cek1 and cAMP-PKA pathways (65, 66). However, contrary to what was expected, both ALS3 and HWP1 were downregulated (Fig. 6B) despite microscopic evidence of robust hyphal formation (Fig. 5). In this regard, Martorano-Fernandes et al. (53) reported functional redundancy among adhesins in mixed biofilms, with ALS1 and HWP1 compensating for reduced ALS3 activity. Furthermore, the work of Hwang et al. (46) also positions ALS3 as having a secondary role in mediating S. *mutans-C. albicans* interactions, which could explain low expression of ALS3. However, similar to what was observed for *S. mutans* gtfB expression, it is possible that *C. albicans* ALS3 and HWP1 were downregulated after 24-h growth in a closed system, and that the overexpression is only observed during exponential growth.

Despite the limitations of this study, our results demonstrate the feasibility of assessing gene expression in caries-relevant oral biofilms after 24-h growth on the bioengineered dentin substrates. All biofilm-related analyses in this study were conducted after 24 h of incubation to evaluate early-stage biofilm formation. Nonetheless, the confined microenvironment, defined by the small diameter of the constructs (5 mm) and the use of a static culture without medium replenishment (likely resulting in early nutrient depletion, potentially well before the 24-h endpoint), may have an impact on the resulting findings (Fig. 6). To capture physiologically relevant gene expression patterns, earlier time points (6–8 h) may be more appropriate, as extended incubation may primarily reflect stress-induced responses or advanced stages of biofilm development (67). Additionally, the use of a static system without medium renewal does not replicate the natural salivary flow, which may affect biofilm dynamics and maturation, particularly during the early phases of biofilm establishment. Furthermore, the dual-species model comprising only *S. mutans* and *C. albicans* oversimplifies the complexity of oral microbial communities, and the use of artificial saliva limits the physiological relevance of biofilm formation. All the above conditions collectively limit the complexity of the current *in vitro* bioengineered construct.

Nevertheless, our construct combining PDMS, collagen, HAP, and surface glycation via MGO aims to represent the most important structural and molecular components of aged dentin. To our knowledge, no existing model integrates all these elements into a single *in vitro* platform suitable for biofilm growth in a static microscale system (Fig. 1). Other groups have also explored the use of PDMS, PMMA, or collagen-based scaffolds to mimic dentin-like structures, primarily within microfluidic or dynamic flow systems (68–71). These studies were mainly designed to investigate dentin–pulp interface processes such as odontoblast activity or cell-material interactions but typically lack one of the principal biochemical constituents of dentin (e.g., collagen or hydroxyapatite) and do not incorporate age-related modifications such as glycation. Other pioneering studies, including the “tooth-on-a-chip” platform developed by França et al. (9, 72) and the microfluidic system proposed by Hu et al. (73), utilize actual human dentin slabs embedded within chips to evaluate either biofilm (*S. mutans*) viability or dental pulp stem cell responses to biomaterials. While physiologically relevant, these models face challenges related to donor variability and limited availability of tooth specimens, which make scalability to high-throughput systems very challenging.

In contrast, our platform provides a reproducible, scalable, and biocompatible environment for investigating early-stage cariogenic biofilm development and virulence. It is compatible with standard 96-well plate formats and adaptable to microfluidic integration, supporting its use in high-throughput applications and biofilm–microenvironment analysis. Current efforts to integrate this bioengineered construct into a microfluidic setup will enhance biological relevance by mimicking dynamic *in vivo* conditions and make the system promising for testing antimicrobial strategies, nutrient modulation, or host-biofilm interactions under normal or aging-related conditions.

### Conclusion

Overall, the bioengineered dentin construct was found to support the growth of 24-h dual-species *S. mutans* and *C. albicans* biofilms, closely resembling the general structure and composition of biofilm formation on aged natural dentin. No significant changes in biofilm biomass, bacterial/fungal ratios, and EPS production were observed between the different surfaces, which suggests that this *in vitro* model is a viable alternative for piloting experiments regarding biofilm formation on aged teeth and reducing the need for human or animal samples for initial proof-of-concept assays. Furthermore, the bioengineered constructs allowed for the exploration of gene expression changes in *S. mutans* and *C. albicans* biofilms as a function of environmental conditions, such as the presence/absence of sucrose and polymicrobial interactions. However, further studies incorporating additional variables are necessary to achieve an *in vitro* model that better replicates the actual *in vivo*, clinical scenario. Nevertheless, the development of bioengineered artificial dentin models will offer several advantages for studies regarding dental aging that include enhanced reproducibility, cost reduction, and the mitigation of ethical concerns associated with the excessive use of animal and human samples.
